# Relationship between *Fusobacterium nucleatum* and antitumor immunity in colorectal cancer liver metastasis

**DOI:** 10.1111/cas.15126

**Published:** 2021-09-23

**Authors:** Yuki Sakamoto, Kosuke Mima, Takatsugu Ishimoto, Yoko Ogata, Katsunori Imai, Yuji Miyamoto, Takahiko Akiyama, Nobuya Daitoku, Yukiharu Hiyoshi, Masaaki Iwatsuki, Yoshifumi Baba, Shiro Iwagami, Yo‐ichi Yamashita, Naoya Yoshida, Yoshihiro Komohara, Shuji Ogino, Hideo Baba

**Affiliations:** ^1^ Department of Gastroenterological Surgery Graduate School of Medical Sciences Kumamoto University Kumamoto Japan; ^2^ Department of Surgery National Hospital Organization Kumamoto Medical Center Kumamoto Japan; ^3^ Gastrointestinal Cancer Biology International Research Center for Medical Sciences Kumamoto University Kumamoto Japan; ^4^ Department of Gastroenterological Surgery Cancer Institute Hospital Tokyo Japan; ^5^ Division of Translational Research and Advanced Treatment Against Gastrointestinal Cancer Kumamoto University Kumamoto Japan; ^6^ Department of Cell Pathology Graduate School of Medical Sciences Kumamoto University Kumamoto Japan; ^7^ Program in MPE Molecular Pathological Epidemiology Department of Pathology Brigham and Women's Hospital and Harvard Medical School Boston Massachusetts USA; ^8^ Department of Epidemiology Harvard T.H. Chan School of Public Health Boston Massachusetts USA; ^9^ Department of Oncologic Pathology Dana‐Farber Cancer Institute Boston Massachusetts USA; ^10^ Broad Institute of MIT and Harvard Cambridge Massachusetts USA; ^11^ Cancer Immunology and Cancer Epidemiology Programs Dana‐Farber Harvard Cancer Center Boston Massachusetts USA

**Keywords:** antitumor immunity, colorectal cancer, *Fusobacterium nucleatum*, gut microbiome, liver metastasis

## Abstract

*Fusobacterium nucleatum* has been detected in 8%‐13% of human colorectal cancer, and shown to inhibit immune responses against primary colorectal tumors in animal models. Thus, we hypothesized that the presence of *F*. *nucleatum* might be associated with reduced T cell density in colorectal cancer liver metastases (CRLM). We quantified *F*. *nucleatum* DNA in 181 CRLM specimens using quantitative PCR assay. The densities of CD8^+^ T cells, CD33^+^ cells (marker for myeloid‐derived suppressor cells [MDSCs]), and CD163^+^ cells (marker for tumor‐associated macrophages [TAMs]) in CRLM tissue were determined by immunohistochemical staining. *Fusobacterium nucleatum* was detected in eight (4.4%) of 181 CRLM specimens. Compared with *F*. *nucleatum*‐negative CRLM, *F*. *nucleatum*‐positive CRLM showed significantly lower density of CD8^+^ T cells (*P* = .033) and higher density of MDSCs (*P* = .001). The association of *F. nucleatum* with the density of TAMs was not statistically significant (*P* = .70). The presence of *F*. *nucleatum* is associated with a lower density of CD8^+^ T cells and a higher density of MDSCs in CRLM tissue. Upon validation, our findings could provide insights to develop strategies that involve targeting microbiota and immune cells for the prevention and treatment of CRLM.

## Graphical abstract

*Fusobacterium nucleatum* was detected in eight (4.4%) of 181 colorectal cancer liver metastasis tissue. We found that the presence of *F. nucleatum* was associated with lower CD8^+^ T cell density and greater densities of both myeloid‐derived suppressor cells and tumor‐associated macrophages.

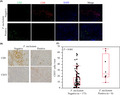

## 1 INTRODUCTION

Colorectal cancer (CRC) is the third most common cancer and second leading cause of cancer‐related death worldwide.^1^ Approximately 50% of patients with CRC will develop liver metastases during the course of disease.^2^, ^3^ However, the 5‐year overall survival time is significantly lower in patients with CRC liver metastases (CRLM) than in those without (16.9% vs 70.4%, respectively).^4^ Hence, there is an urgent need to develop effective strategies for the prevention and treatment of CRLM.

More than 100 trillion bacteria inhabit the human body and form distinct communities in individual organs.^5^ The gut microbiota has recently been shown to play important roles in the immune system and health conditions, including cancer.^6^, ^7^, ^8^, ^9^, ^10^ Metagenomic analyses have revealed the enrichment of *Fusobacterium nucleatum* in primary CRC tissue, which has been confirmed by quantitative PCR (qPCR).^11^ *Fusobacterium nucleatum* is an anaerobic gram‐negative bacterium that has been shown to inhibit antitumor immune responses through the recruitment of myeloid‐derived suppressor cells (MDSCs) and tumor‐associated macrophages (TAMs) into the intestinal tumor microenvironment in animal models.^12^ In previous reports, *F*. *nucleatum* was detected in formalin‐fixed, paraffin‐embedded (FFPE) specimens from 8.6% and 13% of primary CRCs in Japan and the United States, respectively.^13^, ^14^ Greater densities of *F*. *nucleatum* in human primary CRC tissue have been associated with a right‐sided tumor location, high‐level microsatellite instability, and lower T cell density.^14^

The liver is exposed to gut microbiota through the enterohepatic circulation.^15^ The gut microbiota and their metabolites were reported to suppress antitumor immunity in the liver tumor microenvironment.^16^ Bullman and colleagues reported that *Fusobacterium* species were present in CRLM tissues^17^; however, to the best of our knowledge, no studies thus far have examined the relationship between *F*. *nucleatum* and antitumor immunity in human CRLM tissues. We hypothesized that the presence of *F*. *nucleatum* might be associated with lower T cell density in CRLM tissue. A better understanding of the relationship between *F*. *nucleatum* and the immune microenvironment in CRLM could open new opportunities to target the gut microbiota and immunity for the prevention and treatment of CRLM. To test our hypothesis, we examined the presence of *F*. *nucleatum* in relation to the densities of CD8^+^ T cells, MDSCs, and TAMs in human CRLM tissues.

## 2 MATERIALS AND METHODS

### 2.1 Patients and specimens

We analyzed 181 FFPE tissue specimens from consecutive patients with CRLM who underwent curative resection at Kumamoto University Hospital between November 2001 and January 2018. A prospectively maintained database was used to identify the patients; clinical data were collected by reviewing each patient’s medical record. Patients were excluded if they underwent noncurative resection and/or if they had extrahepatic metastasis. Written informed consent was obtained from each subject, and the study procedures were approved by the Institutional Review Board of Kumamoto University.

### 2.2 Preoperative chemotherapy

In total, 94 patients received preoperative chemotherapy. In accordance with the approach in previous reports, preoperative chemotherapy was given to patients with initially unresectable or marginally resectable disease (including patients with concomitant extrahepatic disease in a conversion setting) and to patients with disease that was considered highly malignant (including patients who were diagnosed synchronously, patients with a greater number of tumors, and patients with higher levels of tumor markers) in a neoadjuvant setting.^18^, ^19^

### 2.3 Surgical strategy

The objective of surgery was to remove all detectable lesions with a tumor‐free margin. The type of hepatectomy was based on preoperative findings, intraoperative ultrasonography findings, and the liver functional reserve. Nonanatomical partial hepatectomy was preferred if permitted by the tumor location. Portal vein embolization was undertaken if the tumors were unilobar and the future remnant liver was of an insufficient size. Radiofrequency ablation in combination with hepatectomy was used to treat unresectable tumors or those located deep within the remnant liver to spare the liver parenchyma, as previously described.^18^, ^19^

### 2.4 Postoperative follow‐up

After treatment, all patients received regular follow‐up examinations of imaging studies and an estimation of tumor markers such as serum carcinoembryonic antigen and carbohydrate antigen 19‐9. When recurrence was observed, surgical treatment in combination with chemotherapy was preferred if the overall strategy was considered to be potentially curative.

### 2.5 DNA extraction and qPCR for *F. nucleatum*

Genomic DNA was extracted from FFPE tissue specimens of CRLM using a QIAamp DNA FFPE Tissue Kit (Qiagen). The amount of *F*. *nucleatum* DNA was determined by qPCR. The *nusG* gene of *F*. *nucleatum* and the reference human gene *SLCO2A1* were amplified using custom‐made TaqMan primer/probe sets (Applied Biosystems) as previously reported.^20^ The primer and probe sequences for each Custom TaqMan Gene Expression Assay were as follows: *F*. *nucleatum* forward primer, 5′‐TGGTGTCATTCTTCCAAAAATATCA‐3′; *F*. *nucleatum* reverse primer, 5′‐AGATCAAGAAGGACAAGTTGCTGAA‐3′; *F*. *nucleatum* FAM probe, 5′‐ACTTTAACTCTACCATGTTCA‐3′; *SLCO2A1* forward primer, 5′‐ATCCCCAAAGCACCTGGTTT‐3′; *SLCO2A1* reverse primer, 5′‐AGAGGCCAAGATAGTCCTGGTAA‐3′; and *SLCO2A1* VIC probe, 5′‐CCATCCATGTCCTCATCTC‐3′. Assays were carried out in a 384‐well optical PCR plate. Amplification and detection of DNA were undertaken using a LightCycler 480 Instrument II (Roche) under the following reaction conditions: initial denaturation at 95°C for 10 minutes, followed by 45 cycles of denaturation for 15 seconds at 95°C and annealing/elongation for 60 seconds at 60°C. The quantity of *F*. *nucleatum* DNA in each specimen was calculated as a relative unitless value normalized to the quantity of *SLCO2A1* using the 2^−∆∆^ *^Ct^* method, as previously described.^14^, ^20^

### 2.6 Immunohistochemistry and analysis of immune cells in CRLM tissue

The FFPE tissue was serially sectioned at 3‐5 µm, dewaxed, deparaffinized in xylene, and rehydrated using a graded alcohol series. After the deparaffinization of tissue blocks, antigen retrieval was carried out in antigen retrieval solution using a steamer autoclave at 121°C for 15 minutes or by boiling in a microwave oven for 15 minutes. For CD8, CD33, CD163, FOXP3, Ki‐67, interleukin‐6 (IL‐6), and tumor necrosis factor‐alpha (TNFα) staining assays, the samples were incubated with mouse anti‐CD8 (1:100 dilution, clone C8/144B; Dako Japan), mouse anti‐CD33 (1:100 dilution, clone PWS 44; Leica Biosystems), mouse anti‐CD163 (1:200, clone 10D6; Leica Biosystems), mouse anti‐FOXP3 (1:200, clone 236A/E7; Abcam), Ki‐67 (1:50, clone MIB‐1; Dako Japan), IL‐6 (1:200, clone 1.2‐2B11‐2G10; Abcam), and TNFα (1:50, clone TNF/1500R; Abcam), respectively. To evaluate immune cells (CD8, CD163, FOXP3, and Ki‐67), positive cells in the “tumor margin area” were counted in three separate fields at 200× magnification and their average values were calculated. We evaluated immune cells at the invasive margin of CRLM tissues because previous studies have shown that T cells infiltrate predominantly in the invasive margin of CRLM tissues, and that higher densities of T cells at the invasive margin of CRLM are correlated with better patient survival.^21^, ^22^ The analyses of immune cells were counted manually because the liver tissue could show nonspecific staining areas; accurate evaluation might be difficult with a digital microscope and hybrid cell count software. Nonetheless, we also counted CD8^+^ lymphocytes in the tumor margin areas using a digital microscope (BZ‐X700; Keyence) and hybrid cell count software (BZ‐H3C; Keyence), and found a significant correlation between the manual count and digital count (Spearman’s rank correlation coefficient 0.61, *P* < .0001), suggesting that manual counting methods of immune cells in the current study would be reasonable.

To evaluate inflammatory cytokines (IL‐6 and TNFα), intensity and area of immune activity were each scored 1‐4. Staining intensity was scored according to the following criteria: 1, negative, no staining; 2, weak, light brown; 3, moderate, yellow brown; and 4, strong, brown. Proportion of positive cells was graded as follows: 1, 1%‐25% positive tumor cells; 2, 26%‐50% positive tumor cells; 3, 51%‐75% positive tumor cells; and 4, 76%‐100% positive tumor cells. The staining index (range, 2‐8) was calculated by totaling the staining intensity and area.

### 2.7 Multiplex immunofluorescence

For multiplex immunohistochemical staining, an Opal 4‐Color Fluorescent IHC Kit (PerkinElmer) described above was used with anti‐CD8, anti‐CD33, and anti‐CD163 Abs. In accordance with the manufacturer’s instructions, the CD8 staining protocol was optimized using Opal 470 Fluorophore (red); CD33 and CD163 staining protocols were then optimized using Opal 520 Fluorophore (green). Finally, VECTASHIELD mounting medium with DAPI (Vector Laboratories) was used to stain nuclei.

### 2.8 Statistical analysis

All statistical analyses were carried out using JMP software, version 10 (SAS Institute). Densities of immune cells in CRLM tissue were compared between groups using the Mann‐Whitney *U* test. Clinical features were compared between groups using the χ^2^ test. The Kaplan‐Meier method and log‐rank test were used for survival analysis. Overall survival was defined as the time from the date of surgery to the date of death. Relapse‐free survival was defined as the time from the date of surgery to the date of diagnosis of recurrence.

## 3 RESULTS

### 3.1 *Fusobacterium nucleatum* in CRLM tissues

**FIGURE 1:**
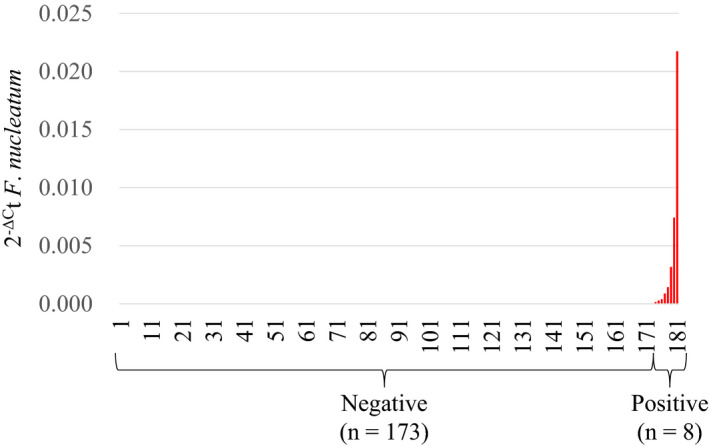
*Fusobacterium nucleatum* in colorectal cancer liver metastasis. Quantity of *F*. *nucleatum* DNA in colorectal cancer liver metastasis tissue

The relative quantities of *F*. *nucleatum* DNA in CRLM tissue specimens were measured by qPCR assays. *Fusobacterium nucleatum* was detected in eight (4.4%) of 181 specimens (Figure 1). Clinical features, stratified according to the presence of *F*. *nucleatum* in CRLM tissue, are shown in Table 1. We did not observe a significant association between tissue *F*. *nucleatum* and serum levels of C‐reactive protein, which is a marker for inflammation (*P* = .75). The presence of *F*. *nucleatum* in CRLM tissue was not significantly associated with age, sex, primary tumor location, timing of CRLM diagnosis, tumor size, tumor number, levels of serum carcinoembryonic antigen and carbohydrate antigen 19‐9, or the use of preoperative chemotherapy. When Kaplan‐Meier analysis was carried out by *F*. *nucleatum* status, *F*. *nucleatum*‐positive cases tended to have a poor prognosis for both overall survival (log‐rank *P* = .15; Figure S1A) and relapse‐free survival (log‐rank *P* = .41; Figure S1B) compared with *F*. *nucleatum*‐negative cases. However, the difference was not statistically significant.

**TABLE 1:** *F*. *nucleatum* DNA status and clinical features

Clinical features	n	*F. nucleatum* DNA		*P*
		Negative	Positive	
All cases	181	173	8	
Mean age ± SD	63.3 ± 11.1	63.5 ± 11.1	58.5 ± 12.0	0.14
Sex				0.38
Male	117	113	4	
Female	64	60	4	
Primary tumor location				0.52
Right	39	38	1	
Left	142	135	7	
Timing of diagnosis				1.00
Synchronous	113	108	5	
Metachronous	68	65	3	
Tumor size				0.84
≦5 cm	154	147	7	
5 cm<	27	26	1	
Tumor number				0.42
<5	135	130	5	
5≦	46	43	3	
CRP (mg/L)	0.36 ± 0.81	0.37 ± 0.06	0.12 ± 0.29	.75
CEA (ng/mL)				0.095
≦3.4	45	45	0	
3.4<	135 (Lack 1)	127 (Lack 1)	8	
CA19‐9 (U/mL)				0.29
≦37	128	121	7	
37<	53	52	1	
Preoperative chemotherapy				0.54
None	87	84	3	
Done	94	89	5	

Abbreviations: CA19‐9, carbohydrate antigen 19‐9; CEA, carcinoembryonic antigen; CRP, C‐reactive protein; SD, standard deviation

### 3.2 Relationship between presence of *F*. *nucleatum* and CD8^+^ T cell density in CRLM tissue

**FIGURE 2:**
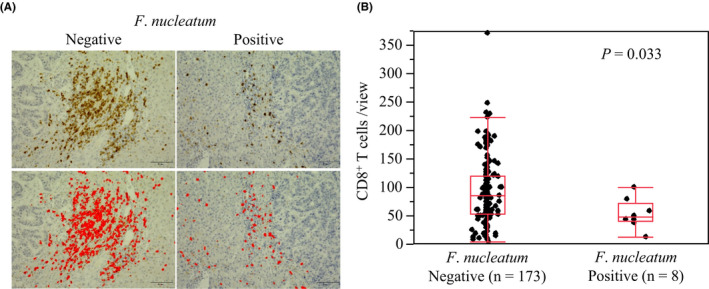
*Fusobacterium nucleatum* and CD8^+^ T cells in colorectal cancer liver metastasis. A, Immunohistochemical staining of CD8 according to the *F*. *nucleatum* DNA status. B, CD8^+^ T cell density according to the *F*. *nucleatum* DNA status

The density of CD8^+^ cytotoxic T cells in CRLM tissue was quantified by immunohistochemistry (Figure 2A). The median follow‐up time for patients with CRLM was 3.4 years. There were 79 deaths among the 181 patients with CRLM. Figure S2 shows the Kaplan‐Meier analyses for overall survival and relapse‐free survival according to CD8^+^ T cell density. Figure S2A,B shows the overall survival and relapse‐free survival after hepatic resection grouping number of CD8^+^ T cells into tertiles. Kaplan‐Meier analysis revealed that a lower CD8^+^ T cell density was significantly associated with shorter relapse‐free survival (log‐rank, *P* for trend = .024; Figure S2B), although there was no significant difference in overall survival according to CD8^+^ T cell density (log‐rank, *P* for trend = .37; Figure S2A). When Kaplan‐Meier analysis was undertaken by stratifying the groups according to low vs high and middle CD8^+^ T cell infiltration levels, the low CD8^+^ T cell infiltration group had significantly shorter relapse‐free survival compared with the high and middle CD8^+^ T cell infiltration group (log‐rank, *P* = .006; Figure S2C). Overall, the presence of *F*. *nucleatum* was significantly associated with a lower density of CD8^+^ cytotoxic T cells in CRLM tissue (*P* = .033; Figure 2B).

We examined expression of Ki‐67 on CD8^+^ T cells in CRLM cases with high CD8^+^ T cell infiltration to investigate activity of CD8^+^ T cells. The positive rate of Ki‐67 expression on CD8^+^ T cells ranged from 1% to 28%, with a median of 5%. There was no significant difference in patient survival according to densities of Ki‐67^+^/CD8^+^ T cells in CRLM cases with high CD8^+^ T cell infiltration (Figure S3).

### 3.3 Relationships between presence of *F. nucleatum* and MDSC and TAM densities in CRLM tissue

**FIGURE 3:**
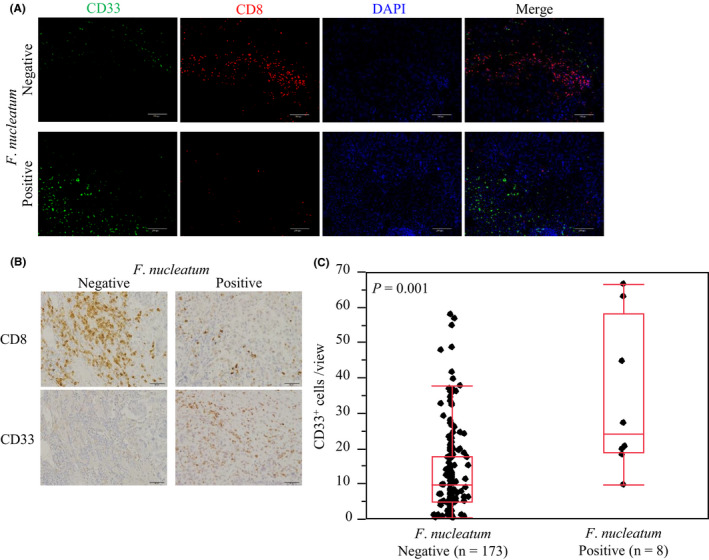
*Fusobacterium nucleatum* and myeloid‐derived suppressor cells in colorectal cancer liver metastasis. A, Multiplex fluorescent immunohistochemical staining of CD8 and CD33. B, Immunohistochemical staining of CD33 according to the *F*. *nucleatum* DNA status. C, Comparison of CD33^+^ cell density according to the *F*. *nucleatum* DNA status

In a mouse model, *F*. *nucleatum* has been shown to suppress the antitumor immune response through the recruitment of MDSCs and TAMs into the intestinal tumor microenvironment through inflammatory cytokines.^12^ Hence, we hypothesized that the presence of *F*. *nucleatum* might be associated with MDSC‐ or TAM‐mediated immunosuppression in CRLM tissue through a mechanism similar to that of the primary colorectal tumor. We compared the densities of CD33^+^ (a marker for MDSCs) and CD163^+^ (a marker for TAMs) cells between *F*. *nucleatum*‐positive CRLM specimens and *F*. *nucleatum*‐negative CRLM specimens. Figure 3A shows the results of immunohistochemical and multiplex fluorescent immunohistochemical staining for CD33^+^ cells. Compared with *F*. *nucleatum*‐negative CRLM specimens, *F*. *nucleatum*‐positive CRLM specimens showed significantly greater density of CD33^+^ cells (*P* = .001; Figure 3B,C). However, there was no significant difference in CD163^+^ cells (*P* = .70; Figure S4).

We also examined the density of FOXP3^+^ cells (a marker for regulatory T cells) between *F*. *nucleatum*‐positive CRLM specimens and *F*. *nucleatum*‐negative CRLM specimens. There was no significant correlation between *F*. *nucleatum* status and density of FOXP3^+^ cells (*P* = .63; Figure S5). Furthermore, we examined expression of inflammatory cytokines, such as IL‐6 and TNFα, as reported previously.^12^ There was no significant association between *F. nucleatum* status and inflammatory cytokines (Figure S6).

## 4 DISCUSSION

This study was undertaken to determine whether the presence of *F*. *nucleatum* was inversely associated with CD8^+^ T cell density in CRLM tissue. The results showed that the presence of *F*. *nucleatum* was associated with a lower CD8^+^ T cell density and a higher MDSC densities in CRLM tissue.

In the *Apc^Min/+^* mouse model, *F*. *nucleatum* promotes colonic neoplasia development through the recruitment of MDSCs into the tumor microenvironment. Myeloid‐derived suppressor cells have been shown to inhibit T cell proliferation and induce T cell apoptosis.^23^, ^24^ These lines of experimental evidence are consistent with our finding of the inverse association between the presence of *F. nucleatum* and the density of CD8^+^ T cells in CRLM tissue. There is increasing evidence that CRC and antitumor immunity are associated with tumor molecular characteristics, tumor progression, and patients’ prognosis.^25^, ^26^, ^27^, ^28^ Myeloid‐derived suppressor cells play important roles in downregulation of the antitumor immune response^29^, ^30^, ^31^; they have been shown to suppress antitumor immunity indirectly through the activity of cytokines such as IL‐10 and transforming growth factor‐β.^32^ These lines of experimental evidence are consistent with our findings regarding an inverse association between the quantity of *F*. *nucleatum* and the density of CD8^+^ T cells in CRLM tissue. Our results support that *F*. *nucleatum* might induce MDSCs in CRLM and suppress CD8^+^ T cells. It has been reported that MDSCs suppress CD8^+^ T cells through programmed cell death‐1 (PD‐1)/PD‐1 ligand (PD‐L1),^33^ therefore anti‐PD‐1/PD‐L1 Abs might be more effective for *F*. *nucleatum*‐positive CRLM patients. In our study, there was no significant difference in the density of TAMs or expression of inflammatory cytokines according to *F. nucleatum* DNA status, in part because the tumor immune microenvironment in the liver could be different from the colorectum. Further investigation is needed to examine the underlying mechanisms.

We detected *F*. *nucleatum* DNA in eight (4.4%) FFPE specimens from patients with CRLM. In previous reports, *F*. *nucleatum* DNA was detected in FFPE specimens from 8.6% and 13% of CRCs in Japan^13^ and the United States, respectively.^14^ Regarding CRLM, Bullman et al^17^ reported that *Fusobacterium* species, including *F*. *nucleatum* and other species, were detected in approximately 50% of *Fusobacterium* species‐positive primary CRC specimens by qPCR. *Fusobacterium nucleatum*‐positive CRCs have been associated with a high level of microsatellite instability.^13^, ^14^ The proportion of stage IV CRCs with high microsatellite instability is reportedly approximately 3%‐5%,^34^, ^35^, ^36^, ^37^, ^38^ which might be consistent with our findings regarding the prevalence of *F*. *nucleatum* in CRLM tissue. Our findings need to be validated by further studies.

Oral health, diet, antibiotics, and pro‐ and prebiotics have been shown to influence the composition of intestinal microbiota.^39^ In light of our findings, it would be intriguing for future investigations of CRLM to explore potential influences of oral health, diet, and lifestyle factors, as well as environmental exposures to *F*. *nucleatum* and its immunosuppressive effects; these factors could have important implications for the development of CRLM prevention and treatment strategies that involve targeting microbiota and immune cells.

We acknowledge some limitations in our study. Because FFPE tissue specimens were used, routine histopathology procedures might have influenced the results of qPCR assays for the detection of *F*. *nucleatum* in CRLM tissue. However, technical artifacts, if any, would have presumably biased our results towards supporting the null hypothesis. We recognize that another limitation of our current study is the lack of a widely accepted, standardized evaluation of antitumor immune response against human CRLM. Hence, we evaluated CD3^+^ T cells in addition to CD8^+^ T cells at the invasive margin of CRLM. However, we did not observe a significant association between density of CD3^+^ T cells and patient survival in CRLM. The current study suggests that density of CD8^+^ T cells at the invasive margin of CRLM are associated with antitumor immune response against human CRLM, although these findings need to be validated by further studies. Further studies are also needed to examine expressions of interferon‐γ, Perforin, granzyme B, and the immune checkpoint molecules (PD‐1, CTLA‐4, TIGIT, LAG3, and TIM‐3) on T cells. Another limitation was that our study used a cross‐sectional design. Hence, we cannot exclude the possibility of reverse causation. Although it is possible that immune cells could eradicate *F*. *nucleatum*, experimental evidence indicating an immunosuppressive effect of *F*. *nucleatum* on T cell activity formed a basis for our specific hypothesis. Because no experimental system can perfectly recapitulate the complex nature of human tumors or the human immune system, analyses of human cancer tissue are useful for elucidating the relationship between microbiota and immunity in cancer.

In conclusion, the presence of *F*. *nucleatum* is associated with a lower density of CD8^+^ T cells and a higher density of MDSCs in CRLM tissue. Following validation, our findings could provide insights to develop strategies that involve targeting microbiota and immune cells for the prevention and treatment of CRLM.

## DISCLOSURE

The authors declare no conflict of interest.

## ETHICS APPROVAL AND CONSENT TO PARTICIPATE

This study was approved by the human ethics review committee of the Graduate School of Medicine, Kumamoto University and the study was carried out in accordance with the Helsinki Declaration of 1964.

## Supporting information

Supplementary Material

Figure S1

Figure S2

Figure S3

Figure S4

Figure S5

Figure S6

## Data Availability

The datasets used and/or analyzed during the current study are available from the corresponding author on reasonable request.
