# Supplementary material for: Relationship between Fusobacterium nucleatum and antitumor immunity in colorectal cancer liver metastasis
Source: Cancer Sci. 2021 Sep 23;112(11):4470–7. doi: 10.1111/cas.15126 (PMC8586672; doi:10.1111/cas.15126)
Supplement: Supplementary file 1 — Supplementary Material [file CAS-112-4470-s005.docx]

**Supplementary Fig. 1. *F. nucleatum* status and patients’ prognosis.**

(A) Overall survival after curative resection of colorectal cancer liver metastases, according to *F. nucleatum* DNA status. (B) Relapse-free survival after curative resection of colorectal cancer liver metastases, according to *F. nucleatum* DNA status.

**Supplementary Fig. 2. CD8^+^ T cells and patients’ prognosis.**

(A) Overall survival after curative resection of colorectal cancer liver metastases, grouping CD8^+^ T-cell numbers into tertiles. (B) Relapse-free survival after curative resection of colorectal cancer liver metastases, grouping CD8^+^ T-cell numbers into tertiles. (C) Relapse-free survival after curative resection of colorectal cancer liver metastases between the group with high and middle infiltration of CD8^+^ T cells and the group with low infiltration of CD8^+^ T cells.

**Supplementary Fig. 3. Expression of Ki-67 on CD8^+^ T cells.**

(A) Immunohistochemical staining of Ki-67. (B) Overall survival and relapse-free survival after curative resection of colorectal cancer liver metastases, grouping number of Ki-67^+^/CD8^+^ T-cell numbers into tertiles.

**Supplementary Fig. 4. *F*. *nucleatum* and tumour-associated macrophages in colorectal cancer liver metastasis.**

(A) Multiplex fluorescent immunohistochemical staining of CD8 and CD163. (B) Immunohistochemical staining of CD163 according to the *F*. *nucleatum* DNA status. (C) A comparison of CD163^+^ cell density according to the *F*. *nucleatum* DNA status.

**Supplementary Fig. 5. Relationship between *F*. *nucleatum* and regulatory T cells in colorectal cancer liver metastasis.**

A comparison of FOXP3^+^ cell density according to the *F*. *nucleatum* DNA status.

**Supplementary Fig. 6. Relationship between *F*. *nucleatum* and inflammatory cytokines in colorectal cancer liver metastasis.**

(A) Expression of interleukin-6 according to the *F*. *nucleatum* DNA status. (B) Expression of tumour necrosis factor-alpha according to the *F*. *nucleatum* DNA status.

**List of Supporting information**

Figure S1 (*F. nucleatum* status and patients’ prognosis.)

Figure S2 (CD8^+^ T cells and patients’ prognosis.)

Figure S3 (Expression of Ki-67 on CD8^+^ T cells.)

Figure S4 (*F*. *nucleatum* and tumour-associated macrophages in colorectal cancer liver metastasis.)

Figure S5 (Relationship between *F*. *nucleatum* and regulatory T cells in colorectal cancer liver metastasis.)

Figure S6 (Relationship between *F*. *nucleatum* and inflammatory cytokines in colorectal cancer liver metastasis.)
