# Supplementary material for: Relationship between Fusobacterium nucleatum and antitumor immunity in colorectal cancer liver metastasis
Source: Cancer Sci. 2021 Sep 23;112(11):4470–7. doi: 10.1111/cas.15126 (PMC8586672; doi:10.1111/cas.15126)
Supplement: Supplementary file 2 — Figure S1 [file CAS-112-4470-s004.pptx]

## Slide 1
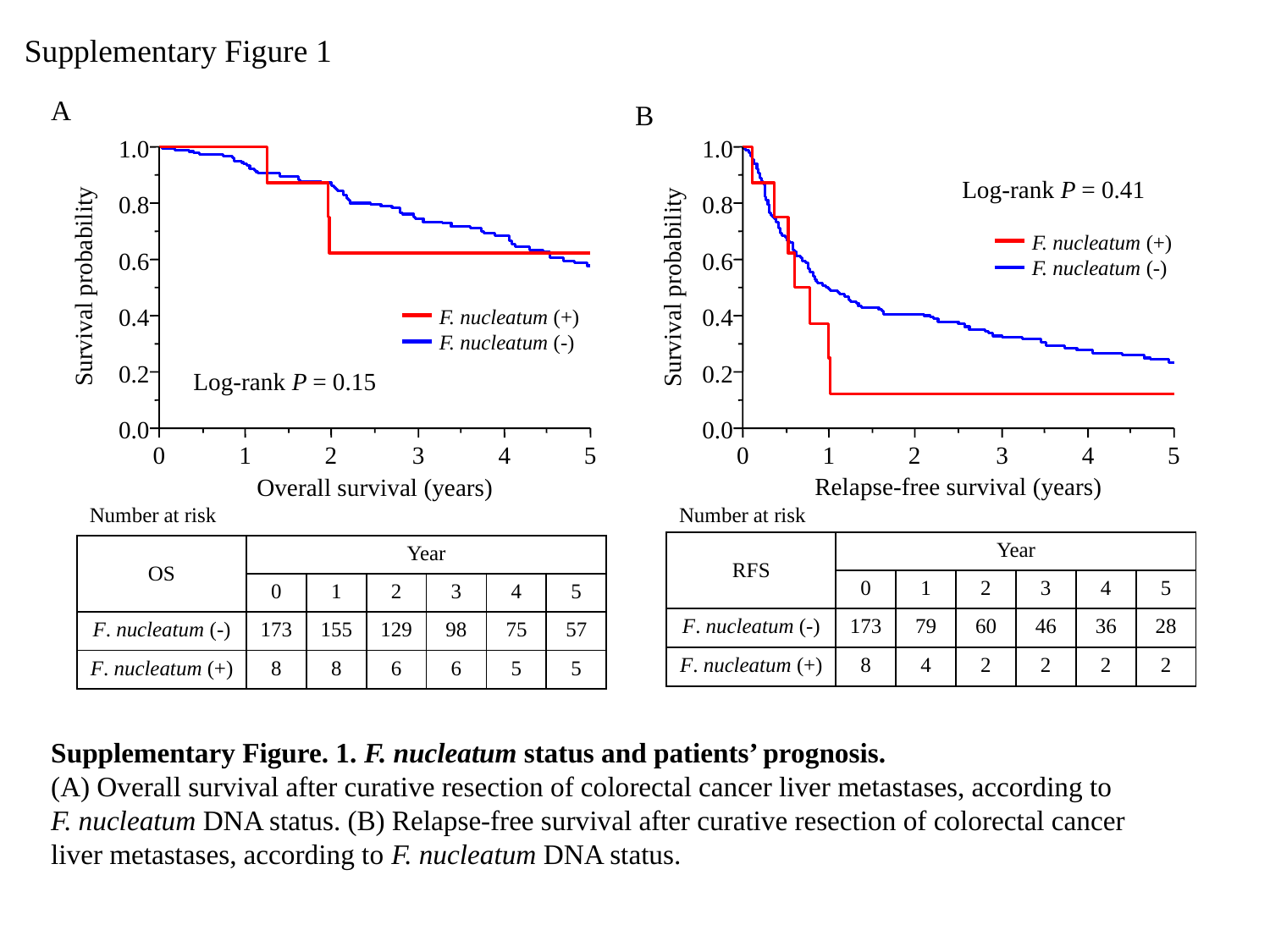

Supplementary Figure 1
A
B
1.0
0.8
0.6
0.4
0.2
0.0
0
1
2
3
4
5
1.0
0.8
0.6
0.4
0.2
0.0
0
1
2
3
4
5
Survival probability
Survival probability
Log-rank P = 0.41
F. nucleatum (+)
F. nucleatum (-)
F. nucleatum (+)
F. nucleatum (-)
Log-rank P = 0.15
Relapse-free survival (years)
Overall survival (years)
Number at risk
Number at risk
| RFS | Year | | | | | |
| --- | --- | --- | --- | --- | --- | --- |
| | 0 | 1 | 2 | 3 | 4 | 5 |
| F. nucleatum (-) | 173 | 79 | 60 | 46 | 36 | 28 |
| F. nucleatum (+) | 8 | 4 | 2 | 2 | 2 | 2 |
| OS | Year | | | | | |
| --- | --- | --- | --- | --- | --- | --- |
| | 0 | 1 | 2 | 3 | 4 | 5 |
| F. nucleatum (-) | 173 | 155 | 129 | 98 | 75 | 57 |
| F. nucleatum (+) | 8 | 8 | 6 | 6 | 5 | 5 |
Supplementary Figure. 1. F. nucleatum status and patients’ prognosis.
(A) Overall survival after curative resection of colorectal cancer liver metastases, according to F. nucleatum DNA status. (B) Relapse-free survival after curative resection of colorectal cancer liver metastases, according to F. nucleatum DNA status.
