# Supplementary material for: Relationship between Fusobacterium nucleatum and antitumor immunity in colorectal cancer liver metastasis
Source: Cancer Sci. 2021 Sep 23;112(11):4470–7. doi: 10.1111/cas.15126 (PMC8586672; doi:10.1111/cas.15126)
Supplement: Supplementary file 3 — Figure S2 [file CAS-112-4470-s003.pptx]

## Slide 1
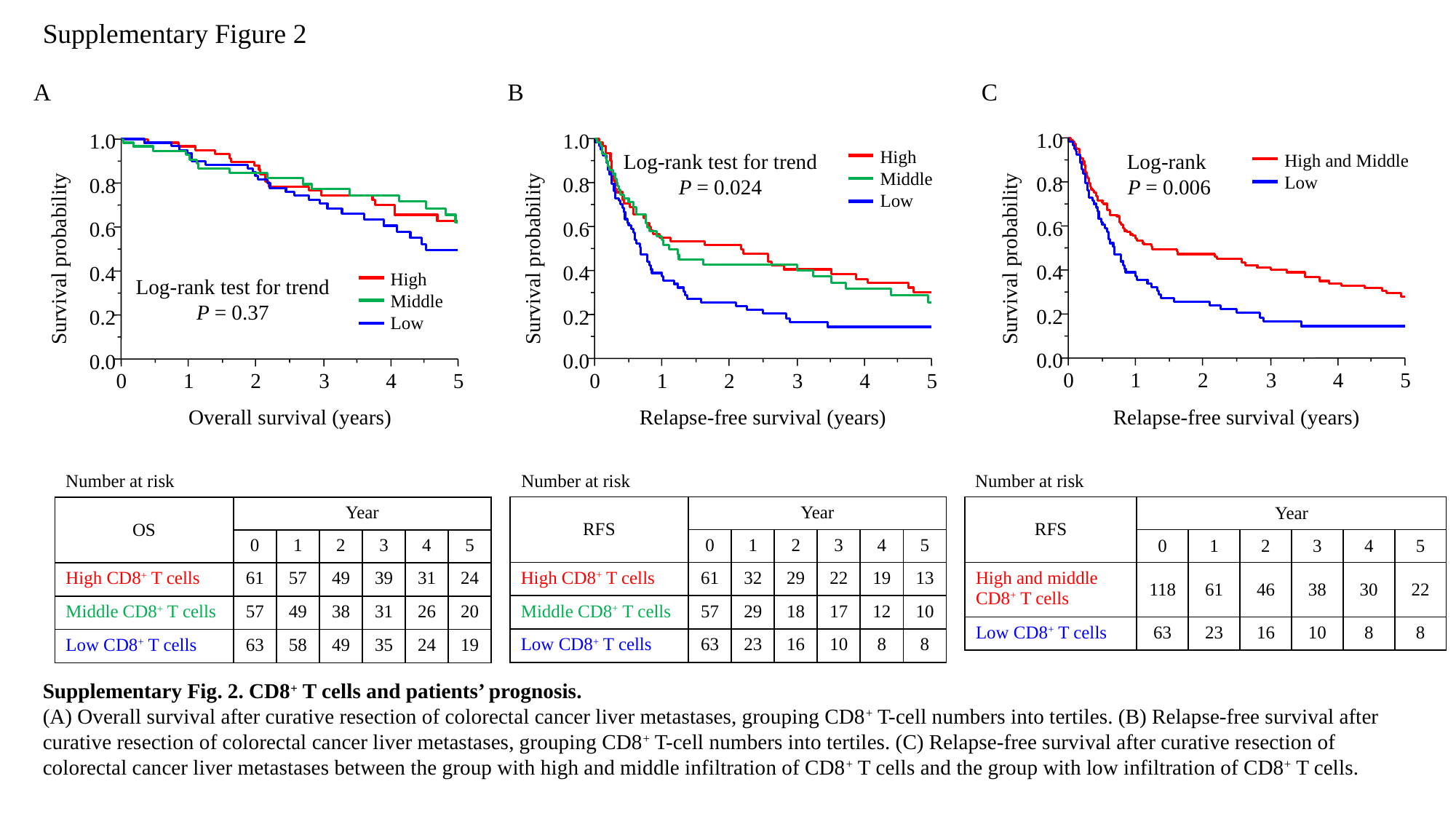

Supplementary Figure 2
A
B
C
1.0
0.8
0.6
0.4
0.2
0.0
0
1
2
3
4
5
1.0
0.8
0.6
0.4
0.2
0.0
0
1
2
3
4
5
1.0
0.8
0.6
0.4
0.2
0.0
0
1
2
3
4
5
High
Middle
Low
Log-rank test for trend
P = 0.024
Log-rank
P = 0.006
High and Middle
Low
Survival probability
Survival probability
Survival probability
High
Middle
Low
Log-rank test for trend
P = 0.37
Relapse-free survival (years)
Relapse-free survival (years)
Overall survival (years)
Number at risk
Number at risk
Number at risk
| RFS | Year | | | | | |
| --- | --- | --- | --- | --- | --- | --- |
| | 0 | 1 | 2 | 3 | 4 | 5 |
| High CD8+ T cells | 61 | 32 | 29 | 22 | 19 | 13 |
| Middle CD8+ T cells | 57 | 29 | 18 | 17 | 12 | 10 |
| Low CD8+ T cells | 63 | 23 | 16 | 10 | 8 | 8 |
| RFS | Year | | | | | |
| --- | --- | --- | --- | --- | --- | --- |
| | 0 | 1 | 2 | 3 | 4 | 5 |
| High and middle CD8+ T cells | 118 | 61 | 46 | 38 | 30 | 22 |
| Low CD8+ T cells | 63 | 23 | 16 | 10 | 8 | 8 |
| OS | Year | | | | | |
| --- | --- | --- | --- | --- | --- | --- |
| | 0 | 1 | 2 | 3 | 4 | 5 |
| High CD8+ T cells | 61 | 57 | 49 | 39 | 31 | 24 |
| Middle CD8+ T cells | 57 | 49 | 38 | 31 | 26 | 20 |
| Low CD8+ T cells | 63 | 58 | 49 | 35 | 24 | 19 |
Supplementary Fig. 2. CD8+ T cells and patients’ prognosis.
(A) Overall survival after curative resection of colorectal cancer liver metastases, grouping CD8+ T-cell numbers into tertiles. (B) Relapse-free survival after curative resection of colorectal cancer liver metastases, grouping CD8+ T-cell numbers into tertiles. (C) Relapse-free survival after curative resection of colorectal cancer liver metastases between the group with high and middle infiltration of CD8+ T cells and the group with low infiltration of CD8+ T cells.
