# Supplementary material for: Relationship between Fusobacterium nucleatum and antitumor immunity in colorectal cancer liver metastasis
Source: Cancer Sci. 2021 Sep 23;112(11):4470–7. doi: 10.1111/cas.15126 (PMC8586672; doi:10.1111/cas.15126)
Supplement: Supplementary file 4 — Figure S3 [file CAS-112-4470-s001.pptx]

## Slide 1
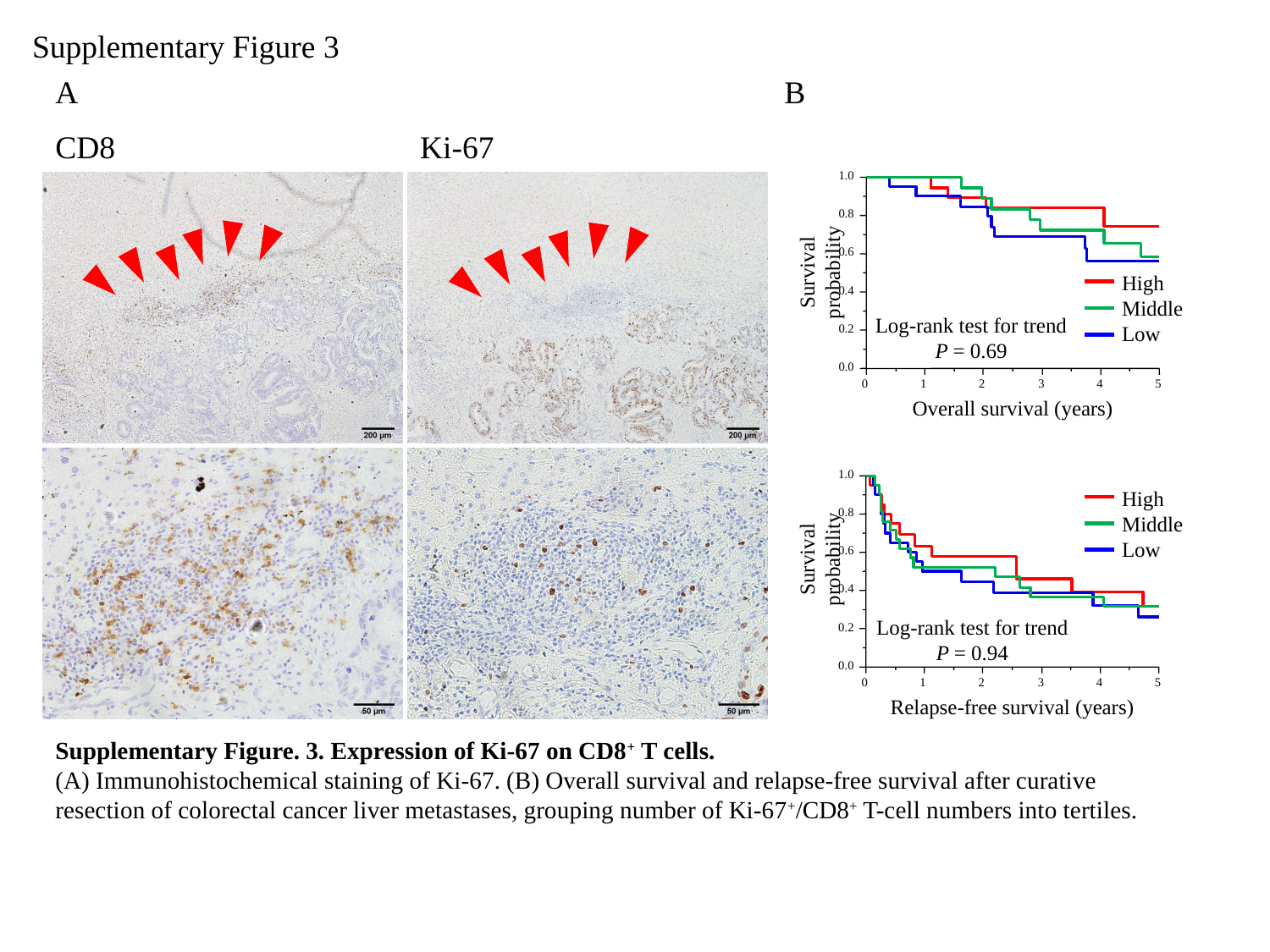

Supplementary Figure 3
A
B
CD8
Ki-67
1.0
Survival probability
0.8
0.6
0.4
0.2
0.0
0
1
2
3
4
5
Overall survival (years)
High
Middle
Low
Log-rank test for trend
P = 0.69
Survival probability
1.0
0.8
0.6
0.4
0.2
0.0
0
1
2
3
4
5
Relapse-free survival (years)
High
Middle
Low
Log-rank test for trend
P = 0.94
Supplementary Figure. 3. Expression of Ki-67 on CD8+ T cells.
(A) Immunohistochemical staining of Ki-67. (B) Overall survival and relapse-free survival after curative resection of colorectal cancer liver metastases, grouping number of Ki-67+/CD8+ T-cell numbers into tertiles.
