# Supplementary material for: Relationship between Fusobacterium nucleatum and antitumor immunity in colorectal cancer liver metastasis
Source: Cancer Sci. 2021 Sep 23;112(11):4470–7. doi: 10.1111/cas.15126 (PMC8586672; doi:10.1111/cas.15126)
Supplement: Supplementary file 5 — Figure S4 [file CAS-112-4470-s002.pptx]

## Slide 1
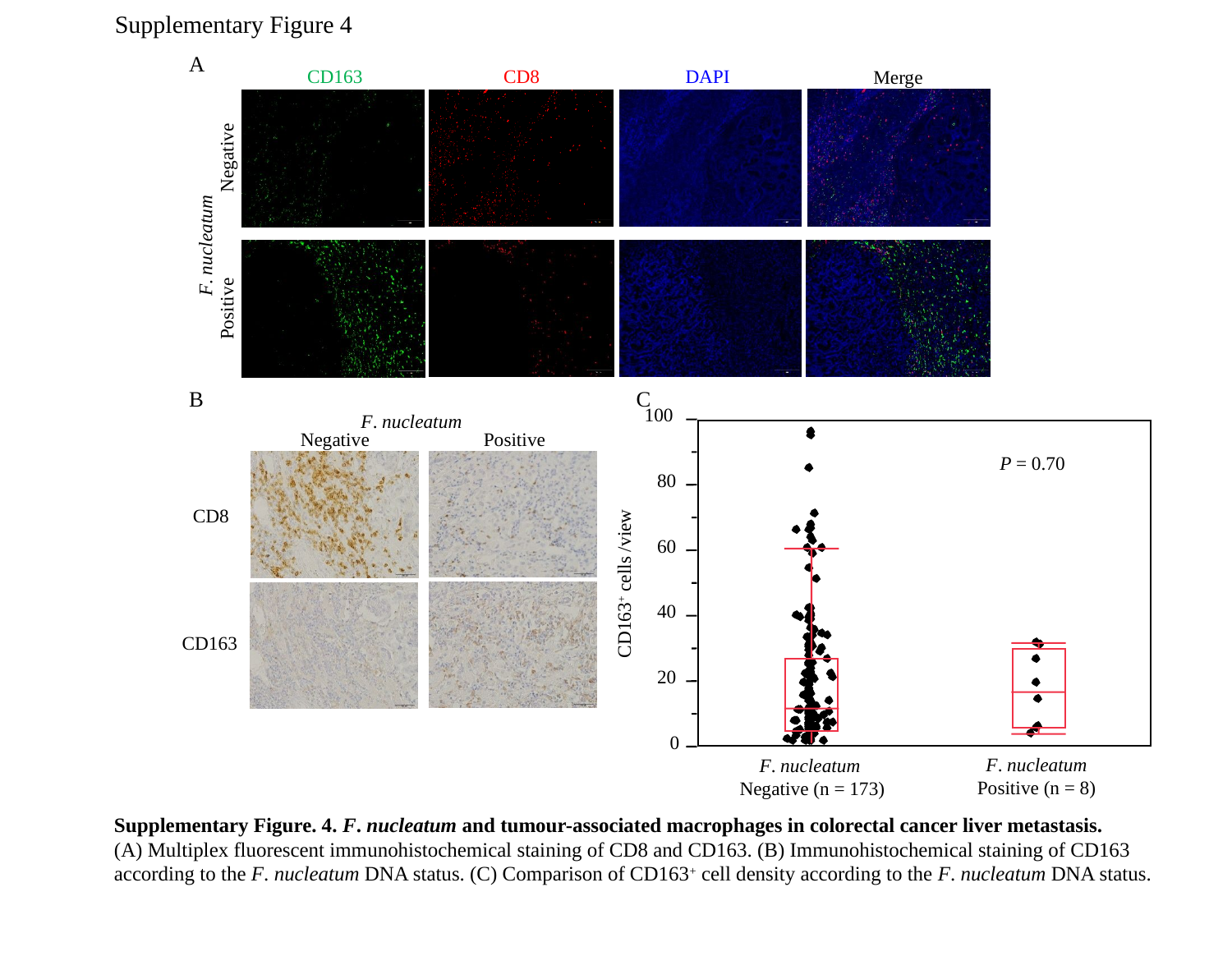

Supplementary Figure 4
A
CD163
CD8
DAPI
Merge
Negative
F. nucleatum
Positive
B
C
100
P = 0.70
80
60
CD163+ cells /view
40
20
0
F. nucleatum
Positive (n = 8)
F. nucleatum
Negative (n = 173)
F. nucleatum
Negative
Positive
CD8
CD163
Supplementary Figure. 4. F. nucleatum and tumour-associated macrophages in colorectal cancer liver metastasis.
(A) Multiplex fluorescent immunohistochemical staining of CD8 and CD163. (B) Immunohistochemical staining of CD163 according to the F. nucleatum DNA status. (C) Comparison of CD163+ cell density according to the F. nucleatum DNA status.
