# Supplementary material for: Relationship between Fusobacterium nucleatum and antitumor immunity in colorectal cancer liver metastasis
Source: Cancer Sci. 2021 Sep 23;112(11):4470–7. doi: 10.1111/cas.15126 (PMC8586672; doi:10.1111/cas.15126)
Supplement: Supplementary file 6 — Figure S5 [file CAS-112-4470-s007.pptx]

## Slide 1
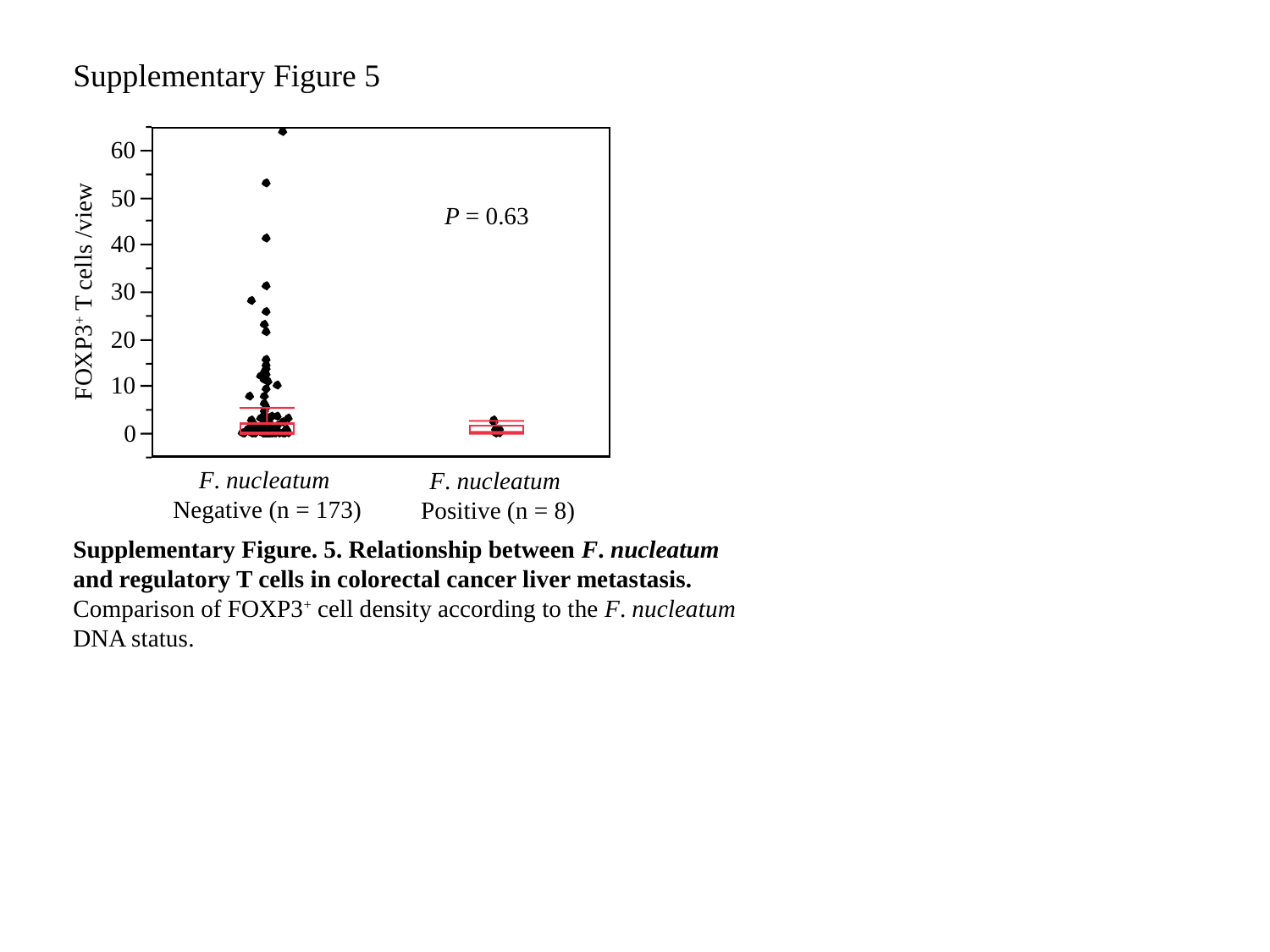

Supplementary Figure 5
60
50
P = 0.63
40
FOXP3+ T cells /view
30
20
10
0
F. nucleatum
Negative (n = 173)
F. nucleatum
Positive (n = 8)
Supplementary Figure. 5. Relationship between F. nucleatum and regulatory T cells in colorectal cancer liver metastasis.
Comparison of FOXP3+ cell density according to the F. nucleatum DNA status.
