# Supplementary material for: Relationship between Fusobacterium nucleatum and antitumor immunity in colorectal cancer liver metastasis
Source: Cancer Sci. 2021 Sep 23;112(11):4470–7. doi: 10.1111/cas.15126 (PMC8586672; doi:10.1111/cas.15126)
Supplement: Supplementary file 7 — Figure S6 [file CAS-112-4470-s006.pptx]

## Slide 1
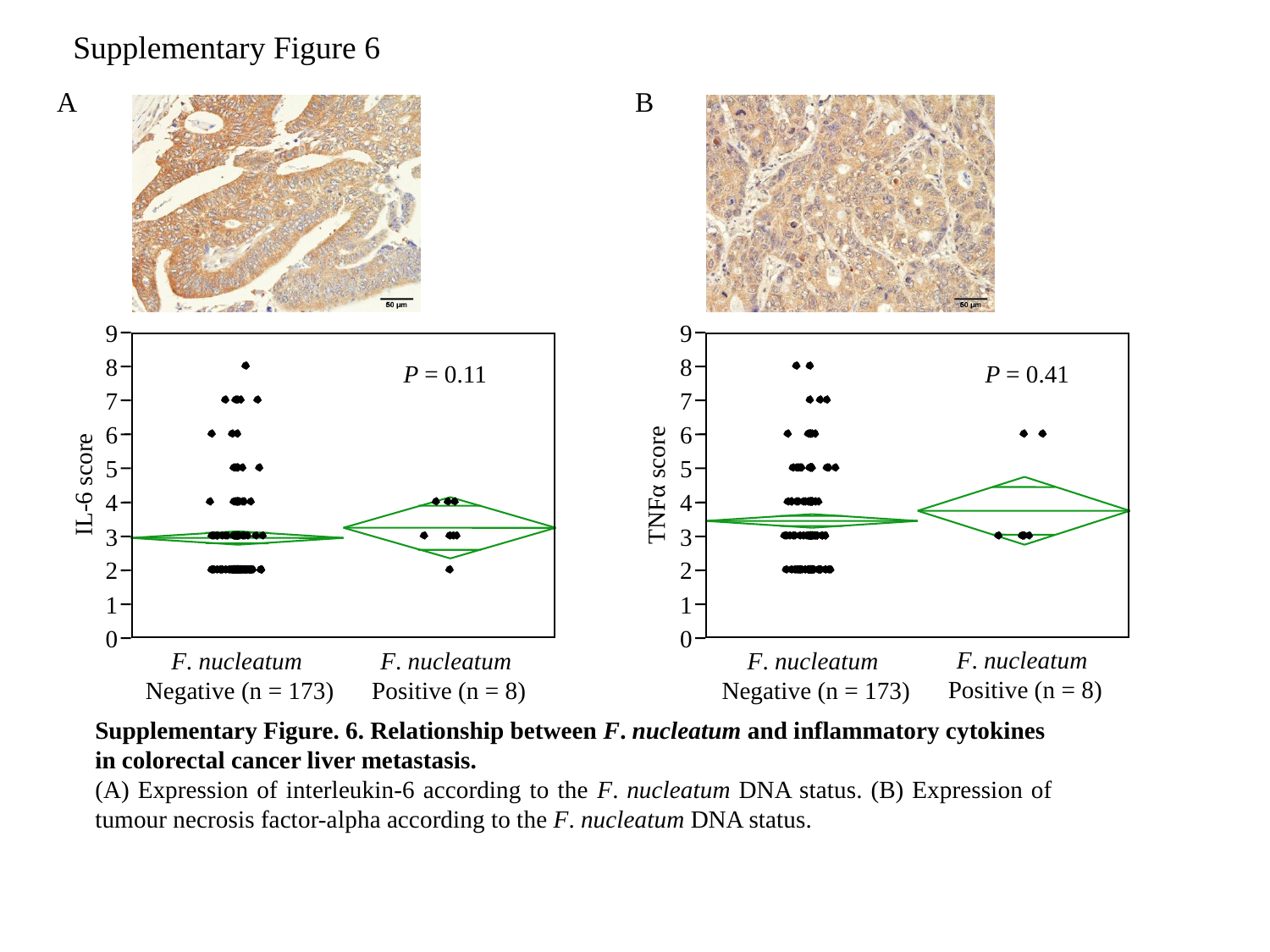

Supplementary Figure 6
A
B
9
8
7
6
5
IL-6 score
4
3
2
1
0
F. nucleatum
Positive (n = 8)
F. nucleatum
Negative (n = 173)
9
8
7
6
5
TNFα score
4
3
2
1
0
F. nucleatum
Positive (n = 8)
F. nucleatum
Negative (n = 173)
P = 0.41
P = 0.11
Supplementary Figure. 6. Relationship between F. nucleatum and inflammatory cytokines in colorectal cancer liver metastasis.
(A) Expression of interleukin-6 according to the F. nucleatum DNA status. (B) Expression of tumour necrosis factor-alpha according to the F. nucleatum DNA status.
